# Characteristics of pathology and transcriptome profiling reveal features of immune response of acutely infected and asymptomatic infected of carp edema virus in Koi

**DOI:** 10.3389/fimmu.2023.1142830

**Published:** 2023-02-27

**Authors:** Ping Ouyang, Yongqiang Ren, Yongheng Zhou, Qiunan Li, Xiaoli Huang, Defang Chen, Yi Geng, Hongrui Guo, Jing Fang, Huidan Deng, Weiming Lai, Zhengli Chen, Gang Shu, Lizi Yin

**Affiliations:** ^1^ Department of Basic Veterinary, College of Veterinary Medicine, Sichuan Agricultural University, Chengdu, Sichuan, China; ^2^ Department of Aquaculture, College of Animal Science and Technology, Sichuan Agricultural University, Chengdu, Sichuan, China

**Keywords:** Koi, carp edema virus (CEV), gill, immune response, RNA-seq

## Abstract

Koi sleepy disease (KSD) is a high mortality and infection viral disease caused by carp edema virus (CEV), which was a serious threat to aquaculture of common carp and export trade of Koi worldwide. Asymptomatic infection is an important cause of the difficulty in preventing KSD and its worldwide spread, because asymptomatic infection can be activated under appropriate condition. However, the understanding of the molecular correlates of these infections is still unknown. The purpose of this study was to compare the pathology change, enzyme activity, immunoglobulin activity, host and viral gene expression differences in acutely infected and cohabiting asymptomatic Koi infected with CEV. Healthy Koi were used as a control. The gross pathology, histopathology and ultrastructural pathology showed the difference and characteristics damage to the tissues of Koi under different infection conditions. Periodic Acid-Schiff stain (PAS), enzyme activity and immunoglobulin activity revealed changes in the immune response of gill tissue between acutely infected, asymptomatic infected and healthy Koi. A total of 111 and 2484 upregulated genes and 257 and 4940 downregulated genes were founded in healthy Koi vs asymptomatic infected Koi and healthy Koi vs acutely infected Koi, respectively. Additionally, 878 upregulated genes and 1089 downregulated genes were identified in asymptomatic vs. acutely infected Koi. Immune gene categories and their corresponding genes in different comparison groups were revealed. A total of 3, 59 and 28 immune-related genes were identified in the group of healthy Koi vs asymptomatic infected Koi, healthy Koi vs acutely infected Koi and asymptomatic infected Koi vs acutely infected Koi, respectively. Nineteen immune-related genes have the same expression manner both in healthy Koi vs acutely infected Koi and asymptomatic Koi vs acutely infected Koi, while 9 immune-related genes were differentially expressed only in asymptomatic Koi vs acutely infected Koi, which may play a role in viral reactivation. In addition, 8 differentially expressed genes (DEGs) were validated by quantitative reverse transcription PCR (RT-qPCR), and the results were consistent with the RNA-Seq results. In conclusion, the data obtained in this study provide new evidence for further elucidating CEV-host interactions and the CEV infection mechanism and will facilitate the implementation of integrated strategies for controlling CEV infection and spread.

## 1 Introduction

Common carp is one of the most economically valuable freshwater fish in global aquaculture according to annual statistics of FAO (Food and Agriculture Organization of the United Nations). The majority of global production of common carp is destined for human consumption, but its ornamental variety, Koi carp, were also considered among most popular ornamental species of fish (1). Intensive culture and international trade of common carp have led to the global spread of virus pathogens of carp, including Koi herpesvirus (KHV), carp edema virus (CEV) and Spring Viraemia of Carp Virus (SVCV) (2–4). Because of the great threat and economic loss to aquaculture caused by these viral pathogens, SVCV and KHV were identified as a disease that were required to be declared by the World Organization for Animal Health (WOAH) (5), and CEV was also included in the Asia-Pacific Aquatic Animal Disease Surveillance List in 2017 (6).

Koi sleepy disease (KSD) is a fatal viral infection caused by Carp edema virus (CEV), which is a DNA double-stranded virus belonging to the poxvirus family (7). This virus had been associated with high morbidity and mortality rates of up to 70–100% (8). CEV was first discovered in Japan in the 1970s (9). Before 2012, it was mainly reported in Japan, and then gradually appeared around the worldwide. KSD has been reported in many countries, including Britain, France, Germany, Italy, India, Korea and China (10–15). It has brought great challenges to carp farming and trade all over the world. The main clinical signs of Koi infected with CEV are lethargy, difficulty in swimming, enophthalmia and swollen and rotten gills, which mainly occur in spring and autumn (4, 16). This virus usually occurs outbreaks at temperatures ranging from 15 to 25°C, but it can also occur when the temperature is below 10°C (8, 17, 18). The mortality rate is relatively lower and the duration is longer during the outbreak of low temperature (4, 19).

The occurrence of CEV had been reported in many places and caused huge economic losses. However, there is no suitable cell line for the culture of CEV at present, which made the isolation and identification of viruses extremely difficult and further hinders the study of pathogenic mechanisms (18). Currently, most of the research on CEV was based on samples from clinical outbreaks. Gills have been confirmed as the main target organs of CEV infection by immunohistochemistry, *in situ* hybridization and real-time fluorescence quantification (20, 21). Gills are not only respiratory organs of fish, but also play an important role in osmotic pressure balance, metabolic function and immune function (22). Metabolomic analysis and blood biochemical indicators showed that the disease was accompanied by a severe disturbance of osmotic pressure balance, which was largely associated with impaired gill respiratory and excretory functions (23). In addition, the significantly down-regulation of CD4, TCRα2 and IgM in gill tissue suggests that complex host-pathogen interactions within the gills can have immunosuppressive consequences (24). However, another study reported that significant upregulation of nine immune-related genes, including IFN-γ, TNF-α, and TGF-β, during CEV infection may induce an antigen-adaptive immune response to inhibits viral replication (25). Although current studies had showed that CEV infection can induce host immune response, there was still little about the mechanism of CEV infection and immune response. Moreover, knowledge of the expression profile and the differences in host genes before and after CEV infection was unknown.

In this study, clinical phenotype, nested PCR, RT-qPCR, histopathology and ultra-pathology were combined to analyze the CEV-infected Koi characteristic pathological injury. The transcriptome was used for the first time to analyze the gene expression in the gill tissue of the Koi under different CEV infection conditions (healthy, asymptomatic infection and acutely infected with obvious symptoms). The common and unique gene expression patterns of acutely infected, asymptomatic infected and clinically healthy Koi indicate the involvement of the modification of host immunity. This will also be beneficial to better understand the pathogenic mechanism of CEV infection and re-activation.

## 2 Materials and methods

### 2.1 Acquisition of Koi samples

In April 2022, persistent deaths in Koi fish farms have been discovered in Chengdu, Sichuan Province. The Koi had lethargy, floating heads, sunken eyeballs, and gill necrosis in the pond. Thirty Koi were randomly selected for parasite, bacteria and virus detection. The livers, spleens, kidneys, gills and other tissues of the Koi were quickly collected and stored in 10% neutral formalin and 2.5% glutaraldehyde for light microscopic and electron microscopic observation. Some tissues were stored at - 80°C for transcriptome analysis, DNA and RNA extraction.

### 2.2 DNA extraction and nested PCR detection

The tissues stored at -80°C were made into tissue homogenate according to the ratio of 1:10 (weight to volume ratio) of normal saline. Viral DNA was extracted from the tissues according to the instructions of TIANamp Genomic DNA Kit (Tiangen Biological Company, China). The purified DNA was stored at -20°C and used for Nested PCR detection (SC/T7229-2019).The primers used in this study were present in **Table 1**. PCR amplifications were performed using ProFlex PCR System (Thermo Fisher Scientific) with total volume of 25 μL containing 12.5 μL 2×PCR Master (Nanjing Vazyme Biotech Co., Ltd), 1 μL of DNA, 1 μL of the forward primer, 1 μL of reverse primer and 9.5 μL of double distilled water. The reaction conditions were used as follows: denaturation at 95°C for 3 min, followed by 30 cycles of 95°C for 15 s, 45°C for 15 s and 72°C for 15 s and elongation at 72°C for 5 min.

**Table 1:** Primers used in this study.

Target gene	Primer name	Sequence (5’-3’)	Site(bp)	Accession No./Reference
Primers for nested PCR				
CEV-P4a	CEV-BF	ATGGAGTATCCAAGTACTTAG	528 bp	(26)
	CEV-BR	CTCTTCACTATTGTGACTTTG		
CEV-P4a	CEV-IF	GTTATCAATGAAATTGTGTATTG	478 bp	(26)
	CEV-IR	TAGCAAAGTACTACCTCATCC		
Primers for Taq-man PCR				
CEV-P4a	CEV-qF1	AGTTTTGTAKATTGTAGCATTTCC	76 bp	(26)
	CEV-qR1	GATTCCTCAAGGAGTTDCAGTAAA		
	CEV-qP1	(6FAM)AGAGTTTGTTTCTTGCCATACAAACT(BHQ1)		
Primers for RT-Qpcr				
IL-1β	IL-1β-F	GCAAGGTCAGGCTGGTCTTATTGT	92 bp	XM_019080073.2
	IL-1β-R	GCTCGGCTTCATCTTGGAGAATGT		
IL-6	IL-6-F	TGAAGACAGTGATGGAGCAGCAGA	104 bp	XM_019110666.2
	IL-6-R	CCTCACAGCAATGTGGCGAACA		
IL-10	IL-10-F	CGCCAGCATAAAGAACTCGT	103 bp	XM_042766262.1
	IL-10-R	TGCCAAATACTGCTCGATGT		
IgM	IgM-F	CACAAGGCGGGAAATGAAGA	170 bp	(24)
	IgM-R	GGAGGCACTATATCAACAGCA		
TNF-α	TNF-α-F	AGGTGATGGTGTCGAGGAGGAAG	122 bp	XM_019088899.2
	TNF-α-R	AGACTTGTTGAGCGTGAAGCAGAC		
CD4+	CD4+-F	CGTGGACATCTGGCTTTGTG	127 bp	(24)
	CD4+-R	TTTGGTTTTGCGTCGTCTGT		
CXCL8a	CXCL8a-F	CGCCTCAAGCATCTAGCAACCAA	168 bp	XM_019099675
	CXCL8a-R	CAATGCAGCGACAGCGTGGAT		
ccl19a.2	ccl19a.2-F	ATTACCGCAGTCCTCTGGAGCAA	198 bp	XM_019104237
	ccl19a.2-R	TTTCTCTGGTGGAGCACACAGTTTC		
IRF3	IRF3-F	TCAACGAGCACCCTAACGAC	139 bp	XM_042715724.1
	IRF3-R	GGACGGATGATGCGATAGAC		
IRF4a	IRF4a-F	CTGAAGGCTGTCGCATCTCC	128 bp	XM_042717694.1
	IRF4a-R	AGACTGAGGGTACGGGAAGG		
C-X-C motif chemokine 10-like	CXC10-F	TGTGCTCTGTGGTTAGGAGTGACT	146 bp	XM_019098792
	CXC10-R	GAGGGACTTGGTGGGAACAATTCAG		
H-2 class II histocompatibility antigen, E-S beta chain	H2-II β-F	CCGAAGATTACACTCAGGTCAGCG	118 bp	XM_042755088.1
	H2-II β-R	TACCATCTCTCAGCCAGGACACTTT		
IL11β	IL11β-F	GTCTACCCTTCCCTTTCACC	116bp	XM_042776482
	IL11β-R	AGGACTCCCAGTGCTCTAAT		
Sting	Sting-F	CAGGTGTGATGGGAGAGGACAGT	103bp	XM_042770656.1
	Sting-R	AGCCAAGCAAACAGCAGAGTAAGAA		
β-action	β-action-F	GACCTGTATGCCAACACTGTAT	209 bp	XM_042720140.1
	β-action-F	TCCTGCTTGCTAATCCACATC		

F, Forward primer; R, Reverse primer.

### 2.3 Quantification of virus genome copies in tissues by real-time TaqMan qPCR

The primers and probes used for real-time TaqMan qPCR were synthesized according to the SC/T7229-2019 (26). The primers were used to amplify fragments of CEV partial 4a gene sequence. The amplicons were cloned into pMD19-T vector (Takara) and propagated in DH5α (Takara). The cloning procedure was following the manufacturer’s directions. The plasmids that successfully expressed the target gene were extracted according to TIANprep Mini Plasmid Kit (Tiangen Biological Company, China). The plasmid bearing the insert was diluted in DNase-free water in a 10-fold dilution series and used to generate a standard curve. The final reaction mixtures contained 2× Master Mix (ChamQ Universal SYBR qPCR Master Mix, Vazyme), 500 nM of each primer, 200 nM of the fluorescent probe and 250 ng of template DNA. The qPCR was performed using a real-time fluorescence quantitative PCR System (Bio-Rad, USA) including an initial denaturation step at 95°C for 5 min, followed by 45 cycles at 95°C for 10 s and 60°C for 30 s. Fluorescence data were analyzed using the real-time fluorescence quantitative PCR System (Bio-Rad). The results were presented as the total number of virus copies per 250 ng of DNA. From a standard curve constructed from 10^1^ to 10^8^ copies of recDNA plasmids of CEV and from aserially (10-fold) diluted CEV-positive sample as a template, we concluded that the qPCR gave the amplification curve y = - 3.32 x + 41.577 with a correlation R2 = 0.999 and efficiency of 100.1% (Range 99.3−105. 1%, **Figure 2A**).

**Figure 1:**
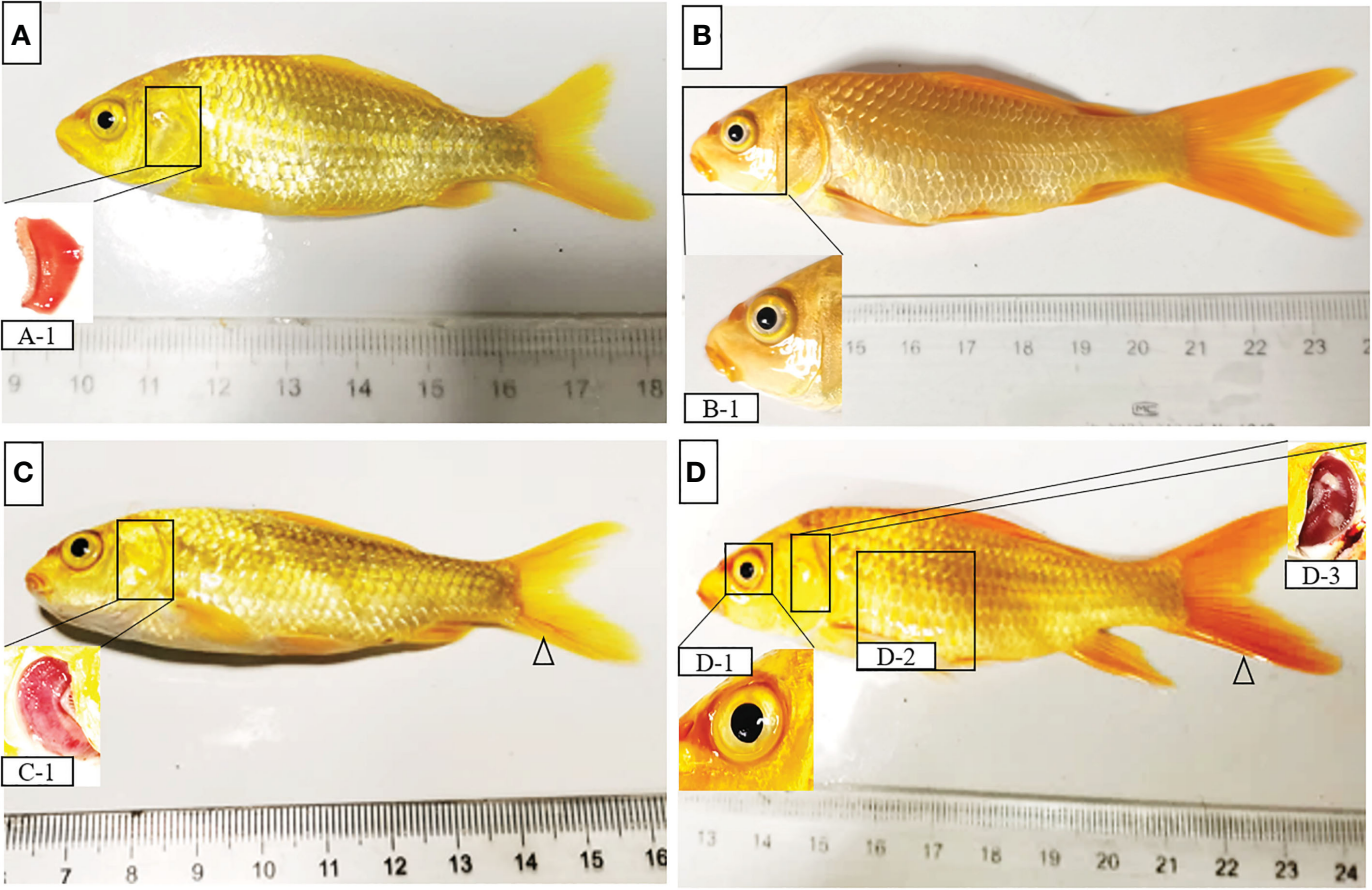
Clinical signs and symptoms of Koi. **(A, B)** Koi had no clinical symptoms and pathology changes **(C)** The gill of sick Koi was necrotic, swollen, mucus increased significantly (C-1), slight bleeding in mandible and caudal fin (△). **(D)** The sick Koi has sunken eyes and slight redness around the eyeball, severe Gill necrosis, mainly characterized by Gill whitening and swelling (D-3), slight abdominal swelling (D-2), caudal fin bleeding (△).

### 2.4 Histopathology and ultramicropathology

The fixed tissue by 10% neutral formalin was washed overnight in running water, dehydrated in a graded ethanol series (50%, 70%, 80%, 90%, 100%× 2), cleaned in xylene, embed in paraffin, subsequently sectioned at approximately 5 μm thickness (LeicaRM2125RTS, Lycra, Germany), and stained with classical hematoxylin and eosin (H&E). The Gill tissue sections were scanned and analyzed by digital pathological scanner (VS120-S6-W (BX61VS)). The length, width and interlamellar area of gill lamella were measured by ImageJ. The Gill tissue samples were fixed with 2.5% glutaraldehyde and 1% osmium tetroxide, dehydrated using graded ethanol (30%→50%→70%→80%→90%→95%→100%×3), thick ultra-thin sections of 50 nm were prepared by ultra-thin section mechanism after infiltration and embedding, stained with uranium acetate and lead citrate, observation and image acquisition using transmission electron microscope (JEM-1400PLUS).

### 2.5 Periodic acid–Schiff staining

The prepared paraffin sections were oxidized 5min with 1% periodate, washed by current water for several times, stained with Schiff reagent, washed by running water, stained with hematoxylin, and sealed with neutral resin after dehydration. The gill tissue sections were scanned by digital pathological scanner (VS120-S6-W (BX61VS)). The number of mucous cells in the same visual field was recorded (0.024 mm^2^).

### 2.6 Enzyme activity analysis

The gill tissue samples were prepared into 10% tissue homogenate according to the ratio of gill weight to normal saline volume of 1:9. The lysozyme (LZM), alkaline phosphatase (AKP), acid phosphatase (ACP), catalase (CAT), glutathione strand hydrogenase (GSH-PX), glutathione (GSH), and superoxide dismutase (SOD) were determined following manufacturer’s instructions (Jian Cheng Bioengineering Institute, Nanjing, China).

### 2.7 ELISA analysis

In order to study the immune response in gill tissue of Koi infected by Carp edema virus at different infection conditions. The contents of related immunoglobulin (IgT and IgM) in gill of Koi was determined by fish immunoglobulin (IgM and IgT) enzyme-linked immunosorbent assay (ELISA) kit (MLBIO Biotechnology Co. Ltd, Shanghai, China) following manufacturer’s instructions.

### 2.8 RNA extraction, cDNA library construction, sequencing and analyses

The total RNA of gill tissue was extracted by Trizol method. Total amounts and integrity of RNA were assessed using the RNA Nano 6000 Assay Kit of the Bioanalyzer 2100 system (Agilent Technologies, CA, USA). The mRNA with polyA tail was enriched by Oligo (dT) magnetic beads, and then the mRNA was randomly interrupted by divalent cations in Fragmentation Buffer, sifted out small fragments of about 370~420 bp. In order to select cDNA fragments of preferentially 370~420 bp in length, the library fragments were purified with AMPure XP system (Beckman Coulter, Beverly, USA). Then PCR amplification, the PCR product was purified by AMPure XP beads, and the library was finally obtained.The clean reads obtained by removing connectors, empty reading, and low quality sequences. The Clean reads were aligned to the reference genome (https://www.ncbi.nlm.nih.gov/genome/?term=Cyprinus+carpio ) using Hisat2 (v2.0.5). DESeq2 software (1.20.0) was used to analyze the differential expression between groups. The unigenes were compared and predicted with known transcripts from six databases to obtain annotated information, including Gene Ontology (GO), Clusters of Orthologous Groups of proteins (COG), Kyoto of Encyclopedia of Genes and Genomes (KEGG), National Center for Biotechnology Information (NCBI) non-redundant protein sequences (NR), Swiss-Prot, and The Pfam protein families (Pfam). The expression level of the transcript was quantitatively analyzed by RSEM software (http://deweylab.github.io/RSEM/ ). Differential expression analysis of two conditions/groups (two biological replicates per condition) was performed using the DESeq2 algorithms. DESeq2 provide statistical routines for determining differential expression in digital gene expression data using a model based on the negative binomial distribution. The resulting P-values were adjusted using the Benjamini and Hochberg’s approach for controlling the false discovery rate. Genes with adjusted P-value <=0.05 and |log2FoldChange|>=1.0 were assigned as differentially expressed.

### 2.9 Validation of differentially expressed transcripts by quantitative real-time PCR

To verify the high-throughput sequencing results from the RNA-Seq data, the upregulated or downregulated genes were detected by RT-qPCR. The primers (**Table 1**) used for RT-qPCR were designed with the Primer Premier 6.0 software. Quantitative real-time PCR assays were performed using a real-time fluorescence quantitative PCR System (Bio-Rad, USA) including an initial denaturation step at 95°C for 5 min, followed by 40 cycles at 95°C for 10 s and 60°C for 30 s. Fluorescence data were analyzed using the real-time fluorescence quantitative PCR System (Bio-Rad). All samples were analyzed in triplicate. The RNA expression of each target gene was normalized to β-actin expression, and the dates was estimated by the 2-^△△^CT method.

### 2.10 Data analysis

In this study, significance analysis was performed using GraphPad Prism 8.0 software (GraphPad Software, La Jolla, CA). The gene expression were normalized by β-action and calculated by 2-^△△^CT method. All experiments were repeated three times at least and the results presented as mean ± SD. The significant level was set as P < 0.05 (*), P < 0.01 (**) , P < 0.001 (***), P < 0.0001 (****).

## 3 Results

### 3.1 Clinical sample analysis by nested PCR and quantitative real-time PCR

**Figure 2:**
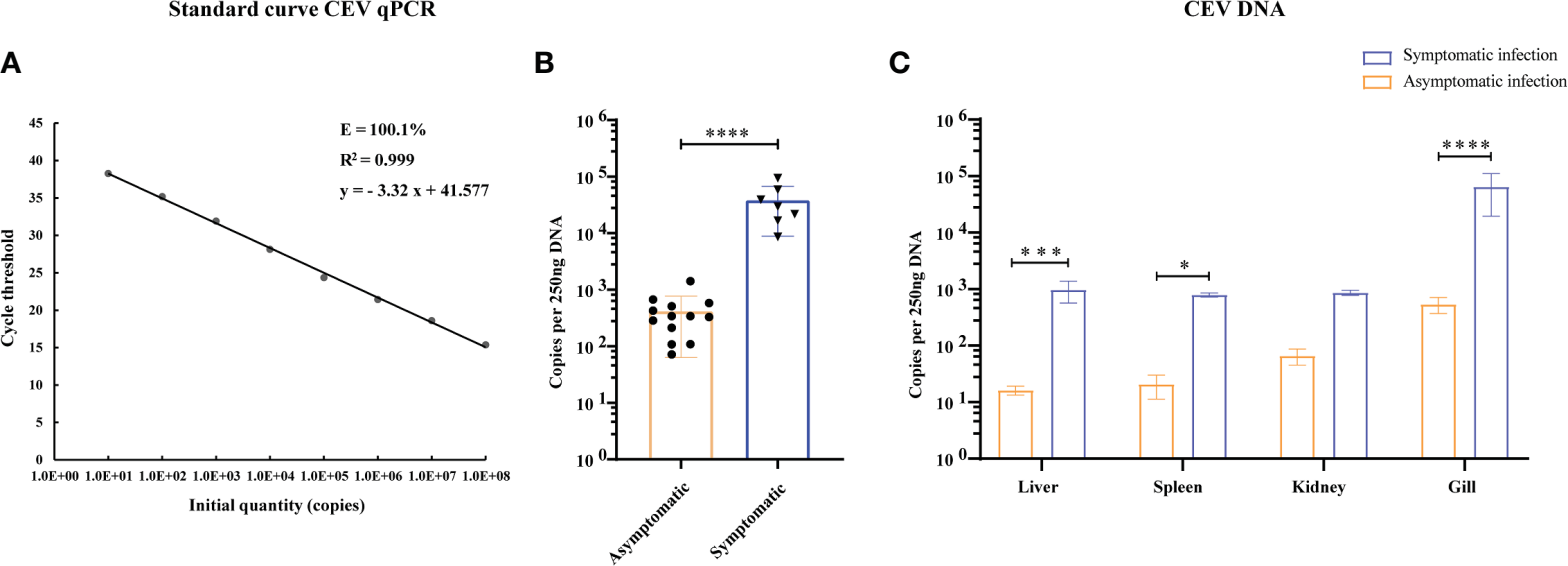
Viral loads in tissues of CEV were analyzed by TaqMan qPCR. **(A)** Standard curve of supercoiled recombinant plasmid DNA from 10^1^ to 10^8^ copies used for real time Taq-man PCR. Standard curve represents mean ± SD from three replicates. Ct (dRn): cycle threshold based on delta (change) of controlized fluorescence; R2: Correlation coefficient; E: efficiency. **(B)** Viral load of CEV in tissues mixture with or without clinical symptoms. **(C)** Viral loads in liver, spleen, kidney and gill tissues of Koi. The results were expressed as the means ± SD of the data observed for the 6 Koi analyzed per 250ng DNA. The significant level was set as P < 0.05 (*), P < 0.001 (***), P < 0.0001 (****).

Thirty Koi were randomly collected for clinical observation and PCR detection, seven Koi showed obvious clinical signs, and the other showed no clinical symptoms. The main clinical signs were exhibited including loss of appetite, lethargy, loss of balance, floating heads, and other external signs such as swollen gills, enophthalmia, and congestion of the caudal fin (**Figure 1**). The fish were negative for parasite and bacterial infection (data not shown). However, nested PCR results showed that some Koi without clinical symptoms were also positive for CEV in tissue mixture, and the proportion was 13/23, although this PCR result was positive only in the second round of nested PCR. In order to further determine whether the asymptomatic Koi carried CEV virus, the viral load of per 250ng DNA in the mixed tissues of liver, spleen, kidney and gill was detected by Quantitative real-time PCR (qPCR). It was found that the Koi without obvious clinical symptoms also carried the virus, and the proportion was as high as 56.5%. However, the viral load of tissues mixtures of asymptomatic Koi was relatively lower than symptomatic Koi (**Table 2**, **Figure 2B**). Through clinical observation, nested PCR and qPCR detection, the Koi without clinical symptoms and negative result of nested PCR were defined as control, and those Koi without clinical symptoms and positive result of Nested PCR for second round were defined as asymptomatic infection, and the Koi with clinical symptoms and positive result of Nested PCR for both round were defined as symptomatic infection. The fish were free of other common viral diseases infection (such as SVCV, Cyprinid herpesvirus 1 (CyHV-1), Cyprinid herpesvirus 2 (CyHV-2) and KHV) were excluded by PCR (data not shown).

**Table 2:** The infection stages of Koi were identified by clinical phenotype, Nested PCR and Taq-man qPCR.

Fish ID	Clinical signs	Nested PCR-F1/R1	Nested PCR-F2/R2	Virus copies
1	No	–	+	213
2	Yes	+	+	29700
3	Yes	+	+	58700
4	Yes	+	+	94000
5	No	–	+	581
6	No	–	+	108
7	No	–	–	–
8	No	–	–	–
9	No	–	+	1420
10	No	–	–	–
11	Yes	–	+	16704
12	No	–	–	–
13	No	–	–	–
14	Yes	+	+	21700
15	No	–	+	342
16	No	–	–	–
17	No	–	+	109
18	No	–	+	672
19	No	–	–	–
20	No	–	+	509
21	No	–	+	342
22	Yes	–	+	8581
23	No	–	+	286
24	Yes	+	+	39300
25	No	–	–	–
26	No	–	–	–
27	No	–	+	72
28	No	–	–	–
29	No	–	+	428
30	No	–	+	329

The clinical symptoms and pathology changes of Koi were observed by three persons. The infection of CEV was confirmed by Nest PCR (+: CEV positive; -: CEV negative). The tissues mixtures were analyzed by real-time TaqMan PCR for quantification of viral genome copies (-: no detected).

### 3.2 Tissue viral load and histopathology injury

In order to study the relationship between the clinical signs, histopathological injury and viral load in the process of CEV infection in Koi, four tissues was analyzed by qPCR. The results showed that the viral load in the tissue of Koi with clinical symptoms was significantly higher than Koi without clinical symptoms (**Figure 2B**). The highest levels of viral DNA were found in the gills in both acutely infected and asymptomatic infection, which indicated that gill was the major target organ of CEV infection. There were also had significant differences of viral load in liver and spleen between acutely infected and asymptomatic infected Koi (**Figure 2C**).

**Figure 3:**
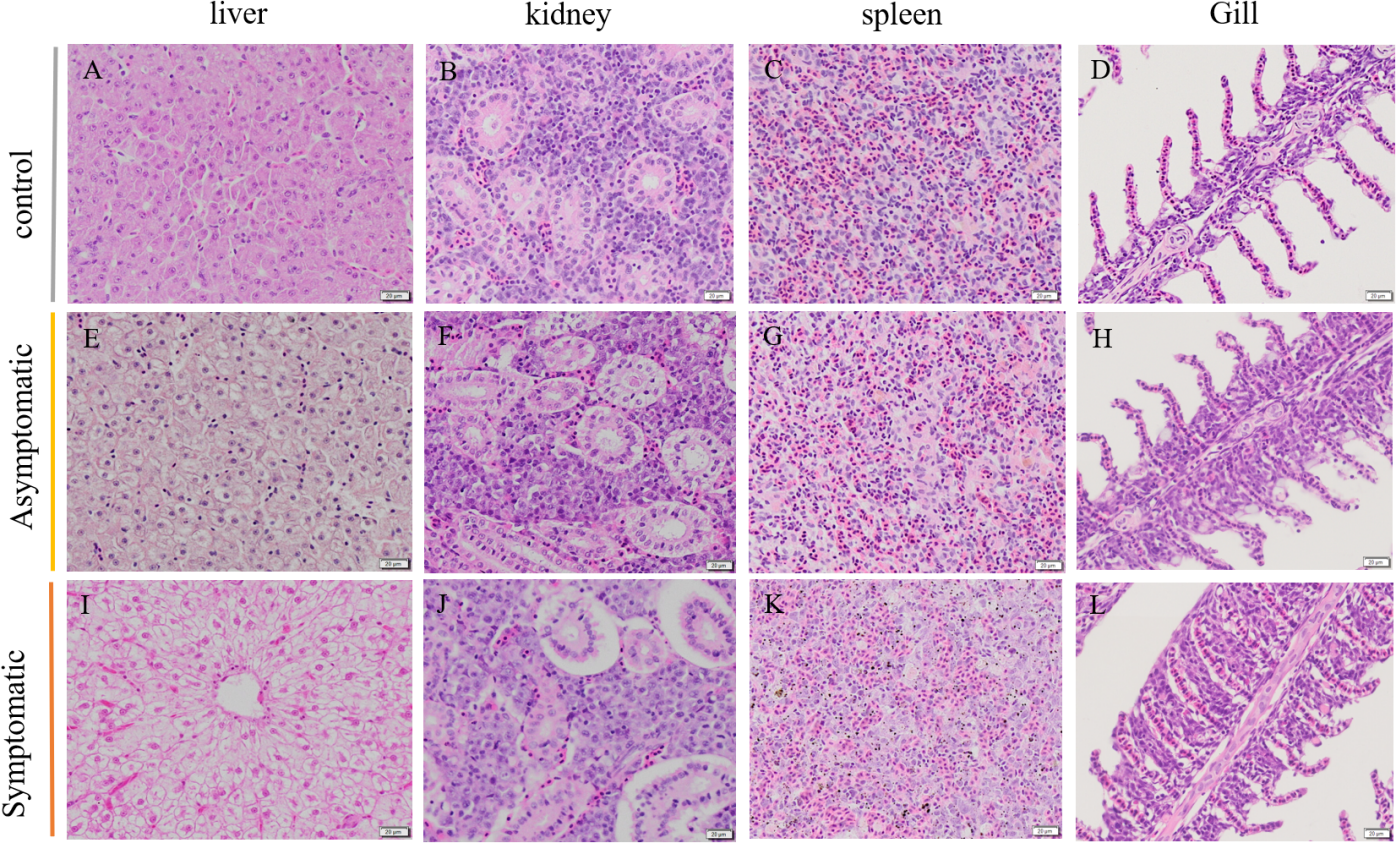
Histopathology change of Koi. **(A-D)** Histological changes in different tissues of control group Koi, **(E-H)** Histological changes in different tissues of asymptomatic infected Koi, **(I-L)** Histological changes in different tissues of symptomatic infected Koi.

**Figure 4:**
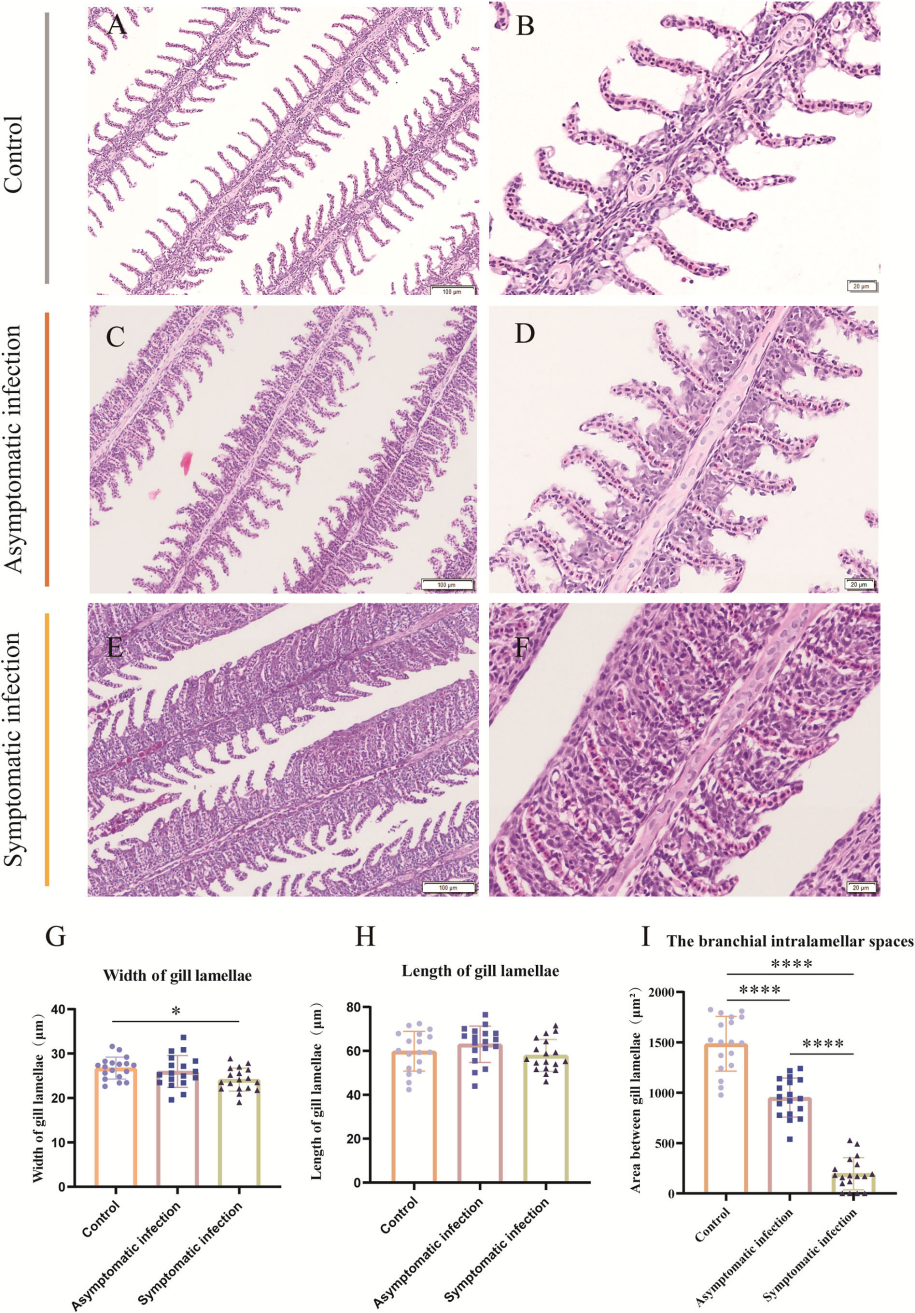
H&E staining, gill lamellae length, gill lamellae width and area between gill lamellae statistics of Koi gill tissues. **(A, B)** The gill of control Koi used as a control. **(C, D)** Gill of asymptomatic Koi with low viral load. **(E, F)** Gill of symptomatic Koi with high viral load. **(G-I)** The length, width of gill lamellae and area between gill lamellae. The significant level was set as: P < 0.05 (*), P < 0.0001 (****), (n =18).

Histopathology results showed that the lesion of gill tissue was the most serious injury organ in both acutely infected and asymptomatic infection Koi (**Figure 3**). The Koi in symptomatic infection group were showed pathology injury in liver, spleen and kidney as well (**Figures 3I-K**). However, the Koi in asymptomatic infection group were showed mild or no pathology injury in liver, spleen and kidney (**Figures 3E-H**). Pathological injure was consistent with the viral load in tissues from acutely infected and asymptomatic infected Koi. The gill lamellae of control Koi were arranged neatly, without swelling, exfoliation and necrosis of epithelial cells (**Figures 4A, B**). Asymptomatic infection Koi had slight proliferation at the base of gill lamellae, accounting for about 1/3 of the whole gill lamellae, epithelial cells swollen and diffusely attached to the gill lamellae and without exfoliation (**Figures 4C, D**). The gill lamellae of Koi with symptomatic infection proliferated obviously, hypertrophy, exfoliation and necrosis of branchial epithelial cells, occlusion of the branchial intralamellar spaces and fusion of secondary lamellae, and even the clav-shaped gill was presented (**Figures 4E, F**). The length, width and area between gill lamella were measured by Image J software. The length of gill lamellae did not change significantly, the width of gill lamellae was significantly different only between control and acutely infected Koi, and the area of branchial intralamellar spaces showed significant difference between each group (**Figures 4G-I**).

### 3.3 Changes of mucous cells in gill tissue

**Figure 5:**
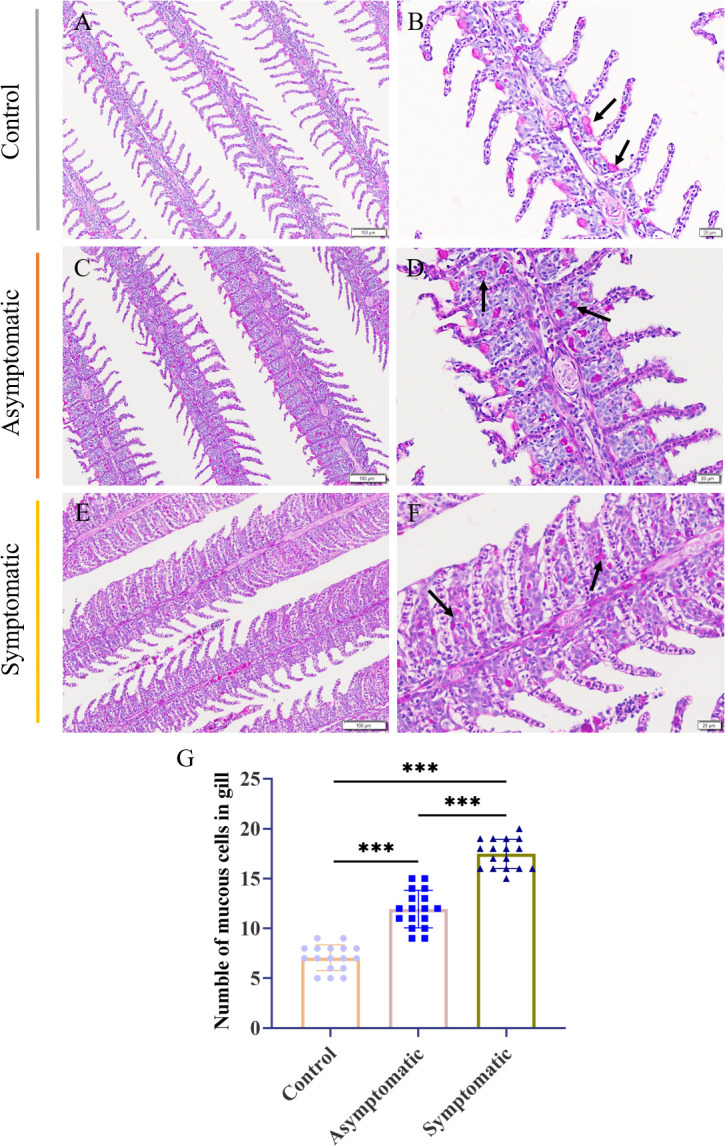
PAS staining and number of mucus cells of Koi gill tissues. **(A, B)** Gill of Koi in the control group. **(C, D)** Gill of asymptomatic Koi with low viral load. **(E, F)** Gill of symptomatic Koi with high viral load. **(G)** The number mucus cells of gill tissues. Significance: P < 0.001 (***) (n =18).

The fish gill was a multipurpose organ, in addition to providing for aquatic gas exchange, plays dominant roles in osmotic and ionic regulation, acid-base regulation, and excretion of nitrogenous wastes (27). Importantly, it was one of peripheral mucosal immune organs of fish. Mucus cells play a crucial role in the immune regulation of gill tissue (22, 28). In order to detect the changes of mucous cells in gill tissue during CEV infection, PAS staining was performed on gill tissue. PAS staining showed that mucus cells of control Koi were attached to the base of gill lamella, and there were one or two mucus cells between each gill lamella (**Figures 5A, B**). Mucous cells of asymptomatic infected Koi migrated laterally from the base of gill lamella (**Figures 5C, D**). The mucous cells were scattered in the branchial intralamellar spaces (**Figures 5E, F**). The number of mucous cells in the gill tissue (0.024 mm^2^) was counted. The result showed that mucous cells increased significantly in the process of CEV infection and the number of mucous cells were the largest in Koi of symptomatic infection (**Figure 5G**).

### 3.4 Electron microscope observation

**Figure 6:**
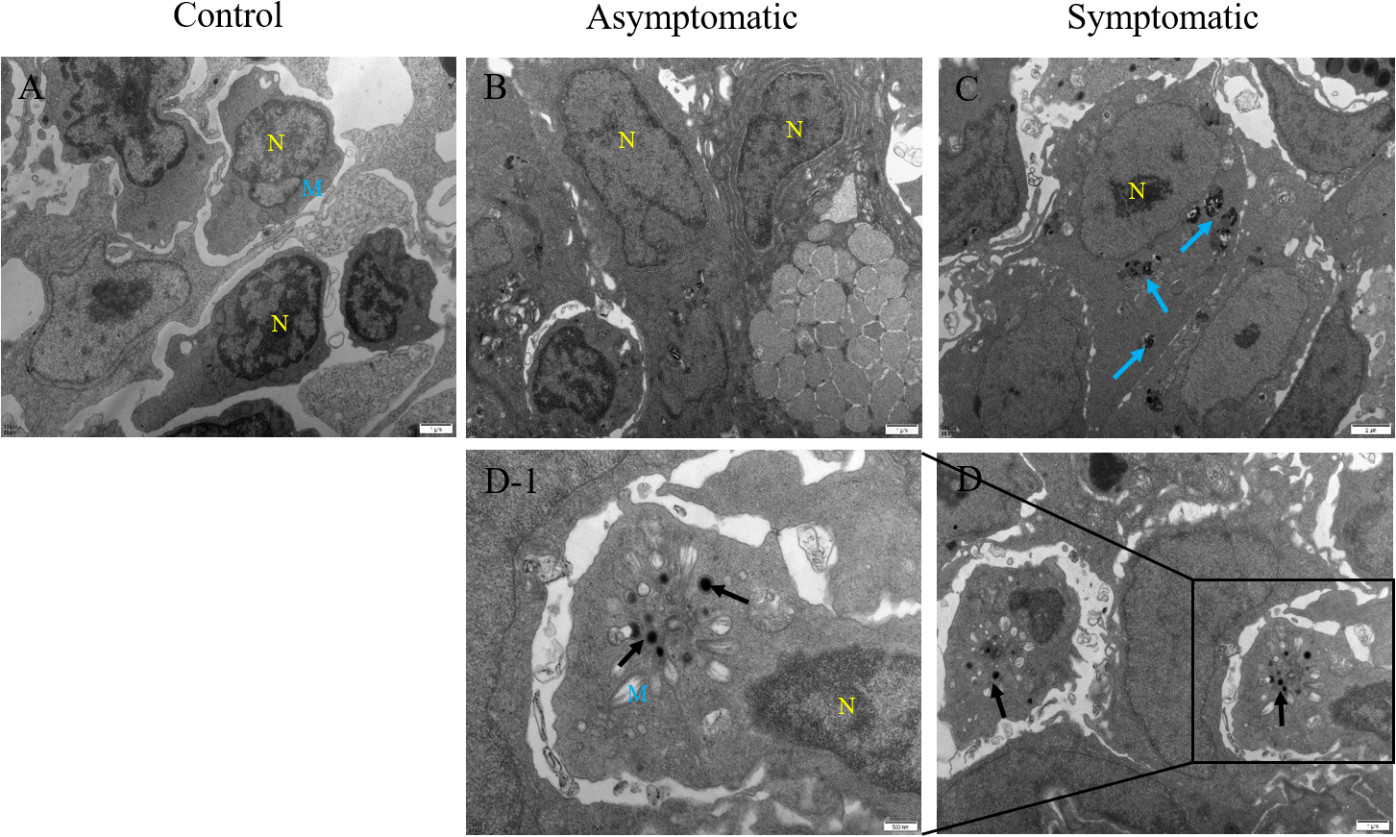
Transmission electron microscope observation of Koi gill tissues. **(A)** Gill of Koi in the control group. **(B)** Gill of Koi in asymptomatic group **(C-D)** Gill of Koi in symptomatic group. N, nucleus; M, mitochondria; Blue arrow, Necrotic cell; Red arrow, virus particles.

To determine the replication process of CEV virions in gill tissue, electron microscope observation was carried out, and the results showed that CEV virions replicate in the cytoplasm and range in size from 200-350nm (**Figure 6**), which was consistent with the results of other studies (16). Healty and asymptomatic Koi had no obvious pathological damage to their gill tissue, the mitochondrial structure was normal and the nucleus was uniformly colored (**Figures 6A, B**). A large number of cell necrosis and obvious mitochondrial swelling were clearly observed in the gill tissue of Koi with clinical symptoms by electron microscope (**Figures 6C, D**).

### 3.5 Enzyme activity and immunoglobulin concentration in gill tissue

**Figure 7:**
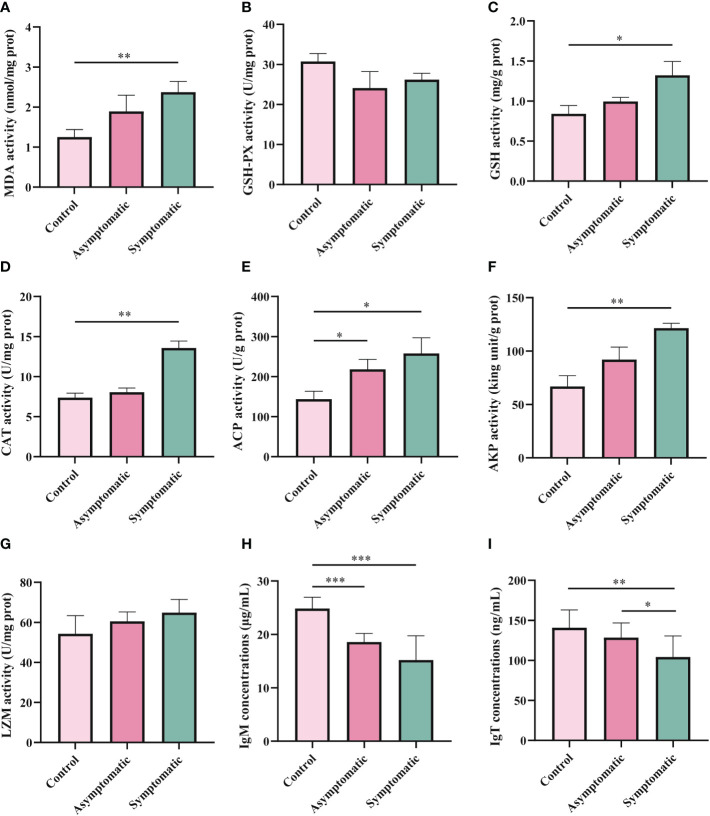
Enzyme activity and activity of immune-related substances were detection in Koi gill tissues. **(A-D)** Oxidative stress activity include catalase (CAT), glutathione strand hydrogenase (GSH-PX), glutathione (GSH), and superoxide dismutase (SOD). **(E-I)** Activity of immune-related substances were detected, include lysozyme, alkaline phosphatase, acid phosphatase, IgM and IgT,respectively. The significant level was set as P < 0.05 (*), P < 0.01 (**) , P < 0.001 (***).

In order to explore the effects of CEV infection on the gill tissue of Koi, enzyme activity and immunoglobulin level in gill tissue were detected in three groups. MDA, GSH-PX, GSH and CAT were detected as oxidative stress indicators, while enzyme activity (ACP, AKP and LZM) and immunoglobulin (IgM and IgT) were detected as immune-related indicators. According to the chemical analysis results, there was significant difference in oxidative stress enzymes activity of SOD, GSH and CAT in gill tissue between control group and symptomatic group Koi, but there was no significant difference between control and asymptomatic Koi (**Figures 7A-D**). The activity of ACP increased significantly than control Koi between asymptomatic infected Koi and acutely infected Koi (**Figure 7E**). The AKP activity increased significantly only between control Koi and symptomatic infected Koi (**Figure 7F**). Although the activity of LZM was higher after CEV infection, there was no significant difference (**Figure 7G**). Conversely, IgM concentrations were significantly decreased in both asymptomatic and symptomatic infected Koi, but IgT concentrations were significantly decreased in symptomatic infected Koi (**Figures 7H, I**).

### 3.6 The expression of immune-related genes in gill tissue

**Figure 8:**
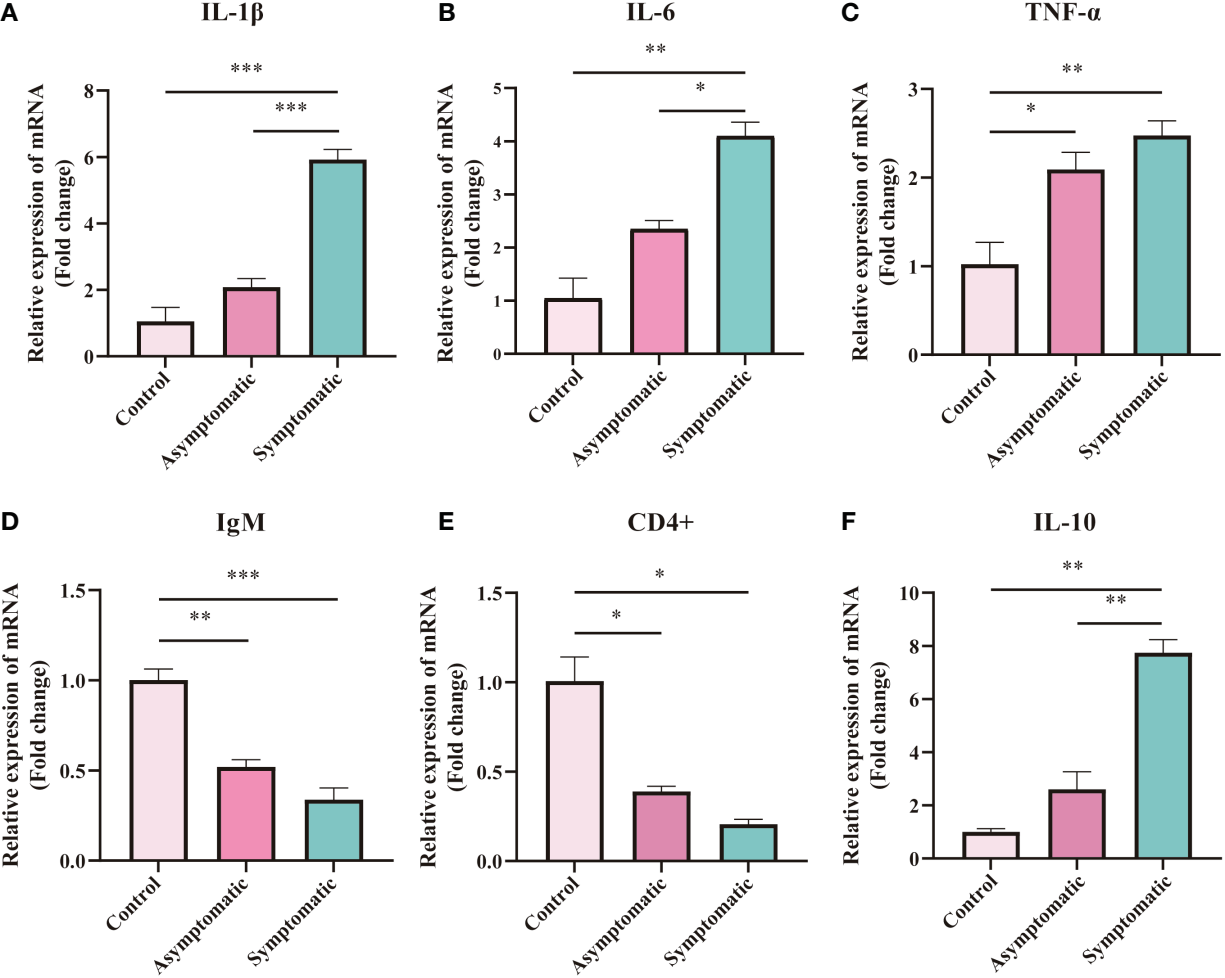
Expression of immune-related gene was measured in gill tissues of Koi at different stages during CEV infection. The RNA expression of each target gene was normalized to β-actin expression. The asterisks (*) indicate significant differences from two groups comparing each other. * indicate P < 0.05, ** indicate P <0.01, ***indicate P <0.001. Data are shown as mean ± SD of six fish.

As the first line of defense against pathogens, gills play an important role in the removal of pathogens (29). In order to investigate the changes of immune-related functions in the gill tissue of Koi during CEV infection, immune-related genes (IL-1β, IL-6, TNF-α, IgM, CD4+ and IL-10) were detected by RT-qPCR. According to the results of RT-qPCR, the expression of TNF-α gene was significantly up-regulated after CEV infection (**Figure 8C**). A significant upregulation was observed in the expression of IL-1β,IL-6 and IL-10 in symptomatic infected Koi compared with control Koi and asymptomatic infected Koi (**Figures 8A, B, F**). On the contrary, the expressions of IgM and CD4+ genes were significantly down-regulated after CEV infection, which consist with the ELISA results of IgM (**Figures 8D, E**).

### 3.7 Transcriptome analysis and gene expression statistics

**Figure 9:**
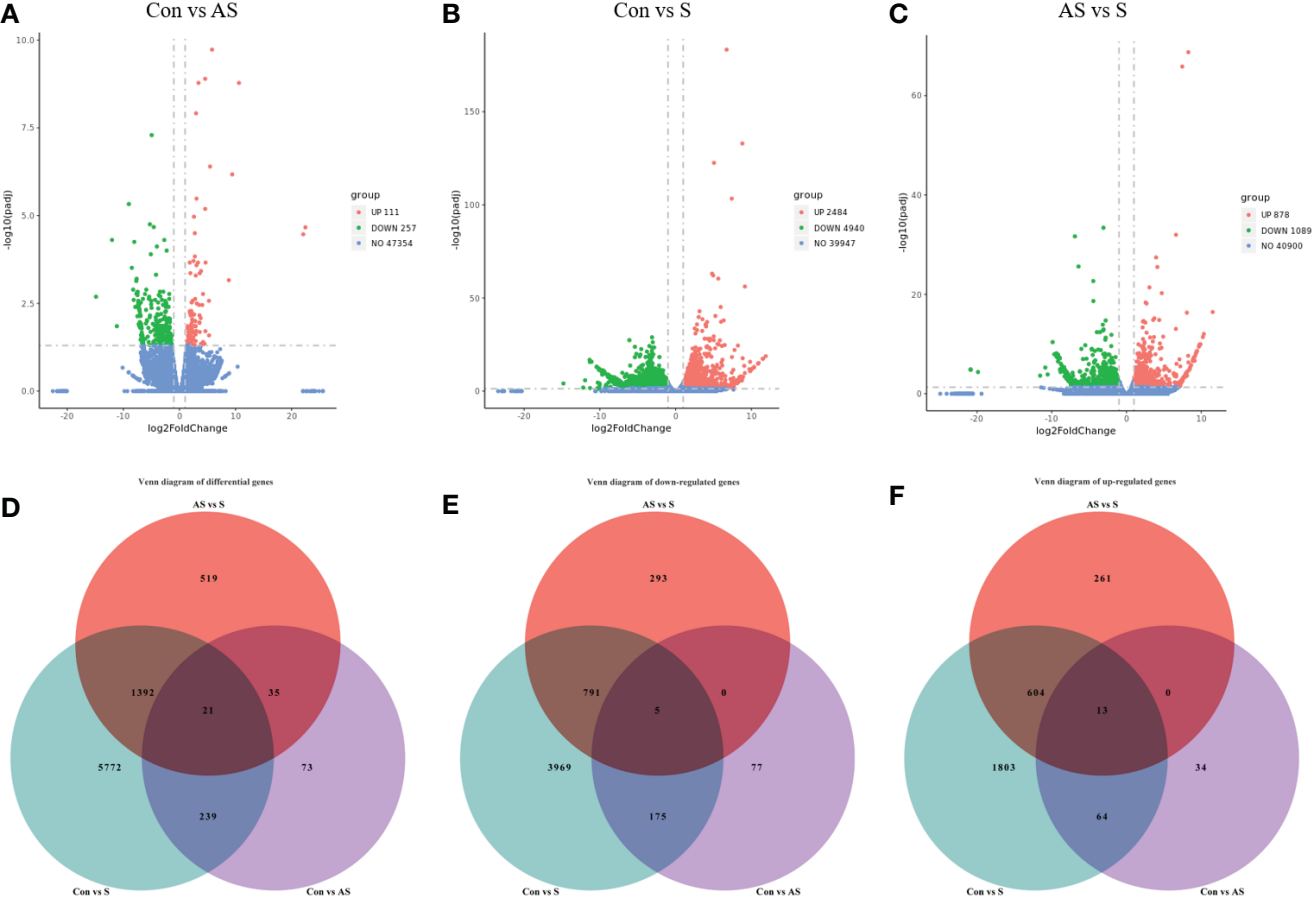
Differentially expressed genes (DEGs) analysis. **(A)** Control group Koi vs. asymptomatic infected Koi volcano plot. **(B)** Control group Koi vs. acutely infected Koi volcano plot. **(C)** Asymptomatic infected Koi vs. acutely infected Koi volcano plot. **(D)** Venn diagram of differentially expressed genes in control group Koi vs asymptomatic Koi, control group Koi vs. acutely infected Koi and asymptomatic infected Koi vs. acutely infected Koi; **(E)** Venn diagram of significantly downregulated genes in control vs asymptomatic Koi, Control group Koi vs. acutely infected Koi and asymptomatic infected Koi vs. acutely infected Koi; **(F)** Venn diagram of significantly upregulated genes in control vs asymptomatic Koi, Control group Koi vs. acutely infected Koi and asymptomatic infected Koi vs. acutely infected Koi.

To investigate the mechanism of gill tissue damage during CEV infection, transcriptome analysis was performed on gill tissue between symptomatic infection, asymptomatic infection and healthy Koi. Nine samples were sequenced by Illumina NovaSeq 6000 sequencing platform, and a total of 42.76 GB of data was obtained, with an average of 4.75GB of data per sample. The clean readings of each sample were compared with the reference genome (https://www.ncbi.nlm.nih.gov/genome/?term=Cyprinus+carpio ), with a comparison rate ranging from 80.73% to 90.25%. Based on analysis, the total number of detected expressed genes was 51290, including 47696 known genes and 3594 predicted novel genes. The differentially expressed genes were analyzed by using variance analysis software DESeq2 (screening threshold for: |log2FC| ≥1, padjust<0.05). The statistical results showed that there were 878 and 2484 upregulated genes and 1089 and 4940 downregulated genes between control Koi vs asymptomatic infected Koi and control group Koi vs symptomatic infected Koi. In addition, the comparisons between asymptomatic infected Koi and symptomatic infected Koi revealed 111 upregulated genes and 257 downregulated genes (**Figures 9A–C**). Furthermore, a total of 21 genes were significantly different in the three comparison groups (**Figure 9D**), 13 genes were significantly up-regulated and 5 genes were significantly down-regulated in all comparison groups (**Figures 9E, F**). (In the co-expressed genes of all the differential genes, there will be cases where the same gene is up-regulated in one comparison group, but down-regulated in the other comparison group, and that gene will be counted in all the Venn diagram co-expressed genes of the differential genes, so the sum of up-regulated and down-regulated genes is different from that of total genes). The RNA-Seq data have been successfully deposited in the SRA database (https://www.ncbi.nlm.nih.gov/sra/PRJNA925573) with accession number PRJNA925573.

### 3.8 GO Analysis of DEGs

**Figure 10:**
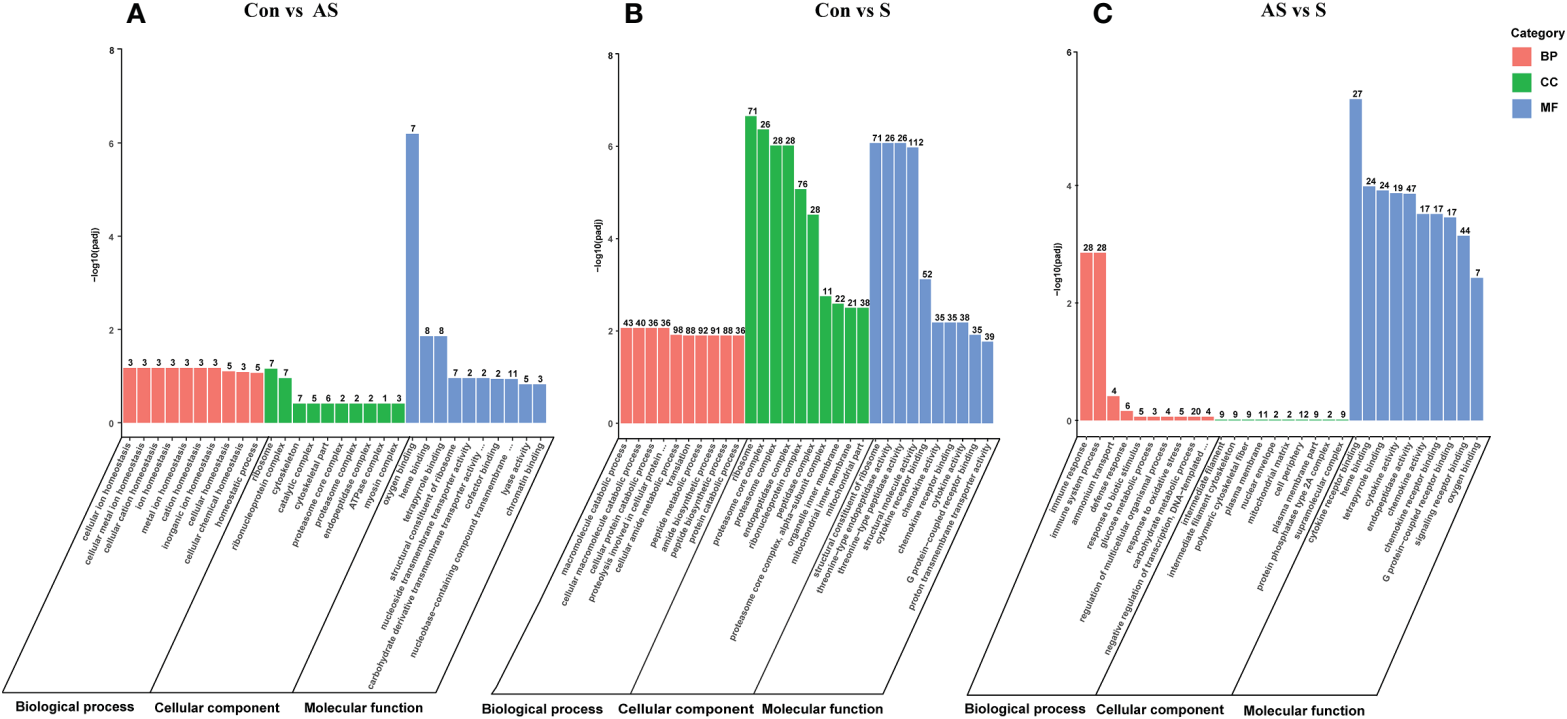
GO classification of DEGs. Biological process, cellular component and molecular function. **(A)** GO analysis of control group vs asymptomatic group. **(B)** GO analysis of asymptomatic group vs symptomatic group **(C)** GO analysis of control group vs symptomatic group.

To analyze the potential biological functions of the DEGs in the three comparative groups, GO annotation of the DEGs was performed according to Molecular Function (MF), Cellular Components (CC) and Biological Process (BP). According to the GO annotation results, the GO terms between control Koi vs asymptomatic infected Koi, control group Koi vs symptomatic infected Koi and asymptomatic infected Koi vs symptomatic infected Koi were 494, 1349 and 956. Ten GO terms with the most significant differences between the comparative groups were listed (**Figures 10A–C**). In this study, the identified MF terms mainly included catalysis, binding activity and molecular biological function, CC terms mainly included cell homeostasis, metabolic process and signal transduction, BF terms mainly included cell and organelle structure and composition.

### 3.9 KEGG analysis of DEGs

**Figure 11:**
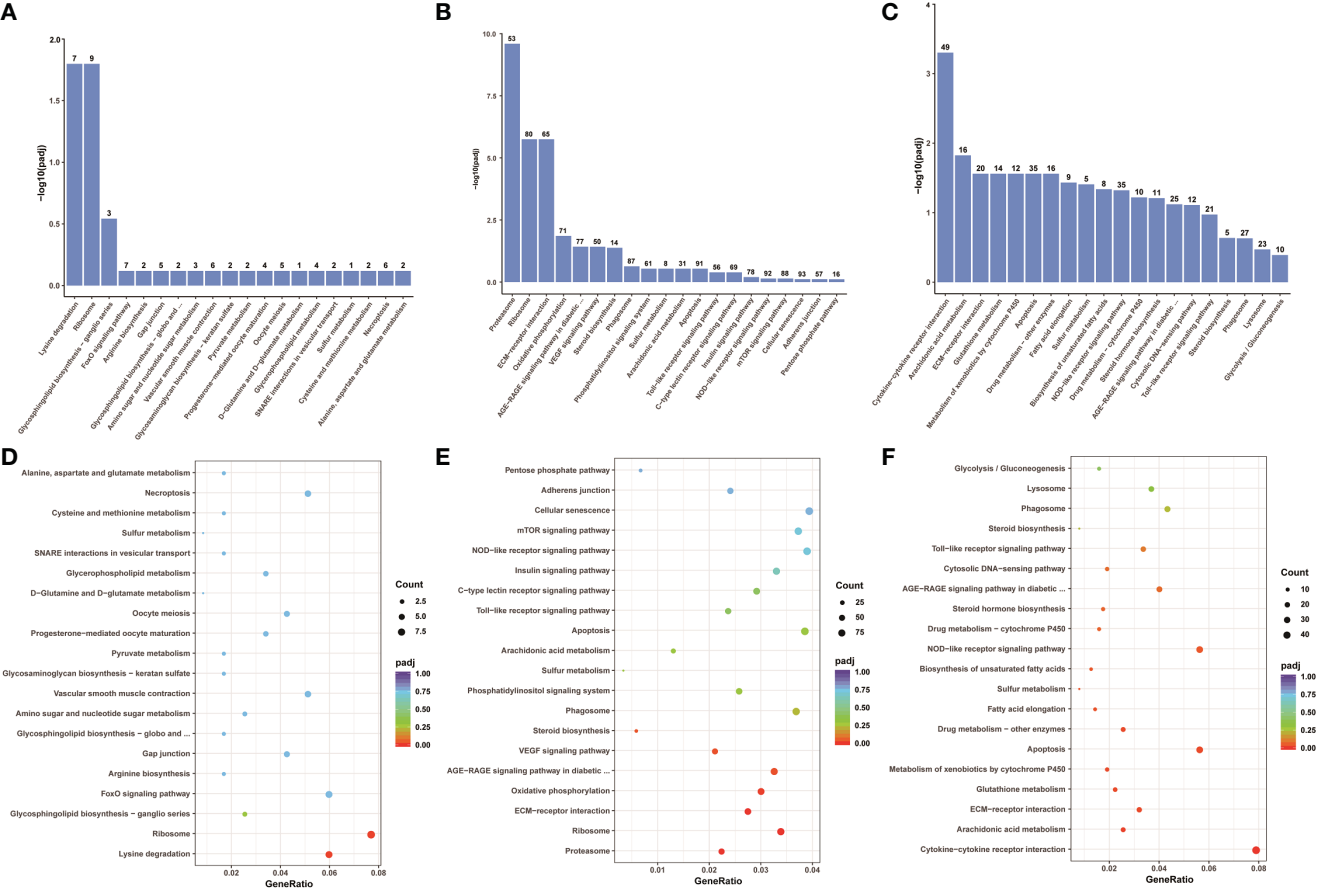
KEGG pathway enrichment analysis of the Koi transcriptome for all comparison groups. **(A, B)** The TOP 20 enriched pathway of DEGs between control group vs asymptomatic group. **(C, D)** The TOP 20 enriched pathway of DEGs between control group vs symptomatic group. **(E, F)** The top 20 enriched pathway of DEGs between asymptomatic group vs symptomatic group.

To explore which signaling pathways were involved in differentially expressed genes, the function of all differentially expressed genes in the KEGG database was annotated. A total of 88 signaling pathways were enriched between control group Koi and asymptomatic infected Koi, the pathways involved in the highest number of differentially expressed genes were mainly Ribosome (ccar03010), Lysine degradation (ccar00310), FoxO signaling pathway (ccar04068), and the differences of the pathways of Ribosome (ccar03010), Lysine degradation (ccar00310) and Glycosphingolipid biosynthesis-ganglio series (ccar00604) were the most significant. A total of 150 signaling pathways were enriched between control group and symptomatic infected group, the pathways involved in the highest number of differentially expressed genes were mainly Cellular senescence (ccar04218), NOD-like receptor signaling pathway (ccar04621), Apoptosis (ccar04210), and the differences of the pathways of Proteasome (ccar03050), Ribosome (ccar03010), ECM-receptor interaction (ccar04512) were the most significant. A total of 144 signaling pathways were enriched between asymptomatic infected Koi and symptomatic infected Koi, the pathways involved in the highest number of differentially expressed genes were mainly Cytokine-cytokine receptor interaction (ccar04060), Apoptosis (ccar04210), NOD-like receptor signaling pathway (ccar04621), and the differences of the pathways of Cytokine-cytokine receptor interaction (ccar04060), Arachidonic acid metabolism (ccar00590) and Apoptosis (ccar04210) were the most significant. The top 20 pathways with the smallest Q values were selected for KEGG enrichment analysis, and most of the signaling pathways were related to immunity (**Figures 11A-F**).

### 3.10 Analysis of immune-related genes and pathways in transcriptome data

**Figure 12:**
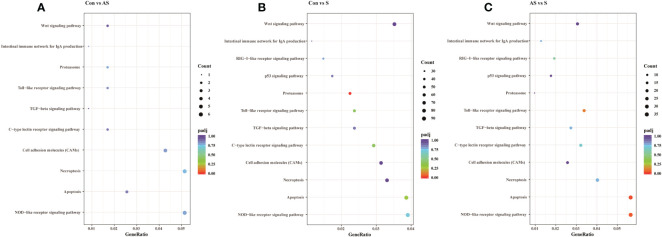
The pathways of immune-related DEG analyzed for the three groups. **(A)** The pathways of immune-related DEG for both comparison Control vs Asymptomatic group. **(B)** The pathways of immune-related DEG for both comparison Control vs Symptomatic group. **(C)** The pathways of immune-related DEG for both comparison group Asymptomatic vs Symptomatic group.

To analyze the mechanism of immunity during CEV infection, the immune-related pathways were screened (**Table 3**), and a bubble chart was used to clarify both the enrichment factors and gene numbers for revealed immune pathways (**Figures 12A-C**). The results showed that the three comparison groups mainly included NOD-like receptor signaling pathway, Toll-like receptor signaling pathway, C-type lectin receptor signaling pathway, Cell adhesion molecules (CAMs), Apoptosis and Necroptosis. In addition, in the comparison groups, among control group Koi vs asymptomatic infected Koi, control group Koi vs symptomatic infected Koi and asymptomatic infected Koi vs symptomatic infected Koi, the numbers of immune-related differential genes were 3, 59, 28, respectively (**Table 4**). The gene of CXC motif chemokine 9-like was significantly up-regulated during CEV infection, and the gene of H-2 class I histocompatibility antigen, Q9 alpha chain was significantly down-regulated. However, most of the genes showed significant difference expression only in the phase of symptomatic infection, the up-regulated genes mainly included cxcl8a, sting1, ccl19a.1, ccl27b and ccl20a.3, and the down-regulated genes mainly included fractalkine-like, CC motif chemokine 20-like, CC motif chemokine 25-like, CC motif chemokine 3-like, H-2 class II histocompatibility antigen, I-E beta chain-like and chemokine (CXC motif) ligand 32b, duplicate 1 (**Table 5**). To reveal the mechanism of immune system of gill tissue during the CEV infection from asymptomatic to symptomatic, the immune-related genes between asymptomatic and symptomatic Koi were compared (**Table 6**). The results showed that there were a total of nine genes upregulated, which mainly included cxcl8a, sting1, ccl19a.1 and ccl19a.2, and a total of 19 genes were down-regulated, including fractalkine-like, H-2 class II histocompatibility antigen, I-E beta chain-like, chemokine (CXC motif) ligand 32b, duplicate 1 and CXC motif chemokine 10-like, most of these genes belong to chemokines, which regulate immune responses by controlling chemotaxis of immune cells and play an important role in the process of CEV infection from asymptomatic to symptomatic.

**Table 3:** The numbers of differentially expressed immune-related genes in the KEGG pathway.

Category	KEGG enriched pathway	Con vs AS		Con vs S		AS vs S	
		Up	Down	Up	Down	Up	Down
Immune-related pathway	NOD-like receptor signaling pathway	3	3	39	53	20	15
	Apoptosis	2	1	44	47	16	19
	Necroptosis	4	2	27	51	13	12
	Cell adhesion molecules (CAMs)	0	5	16	58	4	12
	C-type lectin receptor signaling pathway	1	1	32	37	14	6
	TGF-beta signaling pathway	0	0	10	46	3	14
	Toll-like receptor signaling pathway	2	0	31	25	13	8
	Proteasome	2	0	50	3	1	5
	p53 signaling pathway	0	0	26	15	8	3
	RIG-I-like receptor signaling pathway	0	0	23	12	8	4
	Intestinal immune network for IgA production	0	1	3	24	1	7
	Wnt signaling pathway	0	2	15	8	5	14

**Table 4:** The immunity-related genes of Koi gill tissues in the comparative groups.

Group	Up	Down	Total
Con vs AS	2	1	3
Con vs S	17	42	59
AS vs S	9	19	28

**Table 5:** The expression genes had changed in two comparison groups.

Comparative Groups	Gene ID	Gene symbol	Gene description	log2 (Fold Change)		
				Con vs AS	Con vs S	AS vs S
	**Up (1**)					
Between Con vs AS and Con vs S	109082577		C-X-C motif chemokine 9-like	2.232	3.448	Nf
	**Down(1)**					
	109062092		H-2 class I histocompatibility antigen, Q9 alpha chain	-14.879	-14.801	Nf
Between Con vs S and AS vs S	**Up(6)**					
	109082618		C-C motif chemokine 19	Nf	3.088	2.407
	109085034	cxcl8a	chemokine (C-X-C motif) ligand 8a	Nf	4.057	2.388
	109099637		tumor necrosis factor-like	Nf	2.349	2.321
	109097113	sting1	stimulator of interferon response cGAMP interactor 1	Nf	2.670	2.087
	109090426	ccl19a.1	chemokine (C-C motif) ligand 19a, tandem duplicate 1	Nf	2.472	2.051
	109106190		tumor necrosis factor-like	Nf	1.804	1.921
	**Down(13)**					
	109066054		tumor necrosis factor ligand superfamily member 12-like	Nf	-1.169	-1.344
	109053336		C-C motif chemokine 8-like	Nf	-2.485	-2.089
	109074000		C-C motif chemokine 20-like	Nf	-2.641	-2.365
	109074303		tumor necrosis factor ligand superfamily member 10-like	Nf	-2.331	-2.564
	109045364		interleukin-8-like	Nf	-2.907	-3.427
	109112572		C-C motif chemokine 20-like	Nf	-2.331	-3.731
	109101399		interleukin-8-like	Nf	-3.731	-3.941
	109107897		C-C motif chemokine 25-like	Nf	-4.860	-3.961
	109079521		C-C motif chemokine 3-like	Nf	-3.881	-4.083
	109091496		tumor necrosis factor ligand superfamily member 12-like	Nf	-4.314	-4.460
	109049581		fractalkine-like	Nf	-8.078	-7.921
	109061736		H-2 class II histocompatibility antigen, I-E beta chain-like	Nf	-8.153	-8.153
	109059239		chemokine (C-X-C motif) ligand 32b, duplicate 1	Nf	-8.327	-8.327

**Table 6:** Comparison the genes expression between two groups.

Comparative Groups	Gene ID	Gene symbol	Gene description	log2 (Fold Change)		
				Con vs AS	Con vs S	AS vs S
Con vs AS	novel.151		Unknow	4.459	Nf	Nf
	**Up(10)**					
	122136399		uncharacterized LOC122136399	Nf	6.657	Nf
	109078237	IL-8	interleukin-8	Nf	4.077	Nf
	novel.150		unkown	Nf	2.763	Nf
	109097429		C-C motif chemokine 19-like	Nf	2.429	Nf
	122146497	ccl27b	chemokine (C-C motif) ligand 27b	Nf	2.326	Nf
	109111536	ccl20a.3	chemokine (C-C motif) ligand 20a, duplicate 3	Nf	2.293	Nf
	109046029		monocyte chemotactic protein 1B-like	Nf	1.955	Nf
	109045365		interleukin-8-like	Nf	1.640	Nf
	109088351		uncharacterized LOC109088351	Nf	1.530	Nf
	novel.2381		unkown	Nf	1.194	Nf
	**Down(28)**					
	109111482		phosphoprotein associated with glycosphingolipid-enriched microdomains 1-like	Nf	-1.029	Nf
	novel.1685		Unknow	Nf	-1.296	Nf
	109049583		eotaxin-like	Nf	-1.297	Nf
	109091007		uncharacterized LOC109091007	Nf	-1.481	Nf
	109052935		H-2 class I histocompatibility antigen, Q10 alpha chain-like	Nf	-1.503	Nf
	109064458		serine protease 27-like	Nf	-1.724	Nf
	109072908		tumor necrosis factor ligand superfamily member 14-like	Nf	-1.882	Nf
	109048144		chemokine (C-X-C motif) ligand 12a (stromal cell-derived factor 1)	Nf	-1.962	Nf
	109051707		rano class II histocompatibility antigen, A beta chain-like	Nf	-2.179	Nf
	109053777		C-C motif chemokine 8-like	Nf	-2.287	Nf
	109087419		uncharacterized LOC109087419	Nf	-2.589	Nf
Con vs S	109097460		C-C motif chemokine 3-like	Nf	-2.937	Nf
	109053776		C-C motif chemokine 3-like	Nf	-3.411	Nf
	109086788		zinc-alpha-2-glycoprotein-like	Nf	-3.889	Nf
	109045973		C-X-C motif chemokine 11-6-like	Nf	-4.231	Nf
	122135383		C-C motif chemokine 8-like	Nf	-4.417	Nf
	122145567		growth-regulated alpha protein-like	Nf	-4.617	Nf
	109081322		ectonucleotide pyrophosphatase/phosphodiesterase family member 1-like	Nf	-5.585	Nf
	109046600		somatomedin-B and thrombospondin type-1 domain-containing protein	Nf	-5.716	Nf
	109110796		putative uncharacterized protein DDB_G0282133	Nf	-5.889	Nf
	109095550		C-C motif chemokine 4-like	Nf	-6.027	Nf
	109095323		rano class II histocompatibility antigen, A beta chain-like	Nf	-6.798	Nf
	novel.1543		Unkown	Nf	-6.829	Nf
	109091561		C-C motif chemokine 28-like	Nf	-7.589	Nf
	109093363	ccl36.1	chemokine (C-C motif) ligand 36, duplicate 1	Nf	-7.895	Nf
	109109117		H-2 class II histocompatibility antigen, E-S beta chain-like	Nf	-8.616	Nf
	109111425		uncharacterized LOC109111425	Nf	-8.711	Nf
	122144297		H-2 class II histocompatibility antigen, E-S beta chain-like	Nf	-10.331	Nf
	**Up(3)**					
	109070544		ectonucleotide pyrophosphatase/phosphodiesterase family member 2-like	Nf	Nf	7.021
	109082076		H-2 class II histocompatibility antigen, E-S beta chain	Nf	Nf	6.591
	109090425	ccl19a.2	chemokine (C-C motif) ligand 19a, tandem duplicate 2	Nf	Nf	2.437
AS vs S	**Down(6)**					
	109092916		tumor necrosis factor ligand superfamily member 10-like	Nf	Nf	-1.570
	109063773		interleukin-8	Nf	Nf	-1.713
	109050034		interleukin-8-like	Nf	Nf	-2.736
	109079527		C-C motif chemokine 3-like	Nf	Nf	-3.260
	109073544		uncharacterized LOC109073544	Nf	Nf	-3.939
	109084082		C-X-C motif chemokine 10-like	Nf	Nf	-7.106

### 3.11 Validation of differentially expressed transcripts by RT-qPCR

**Figure 13:**
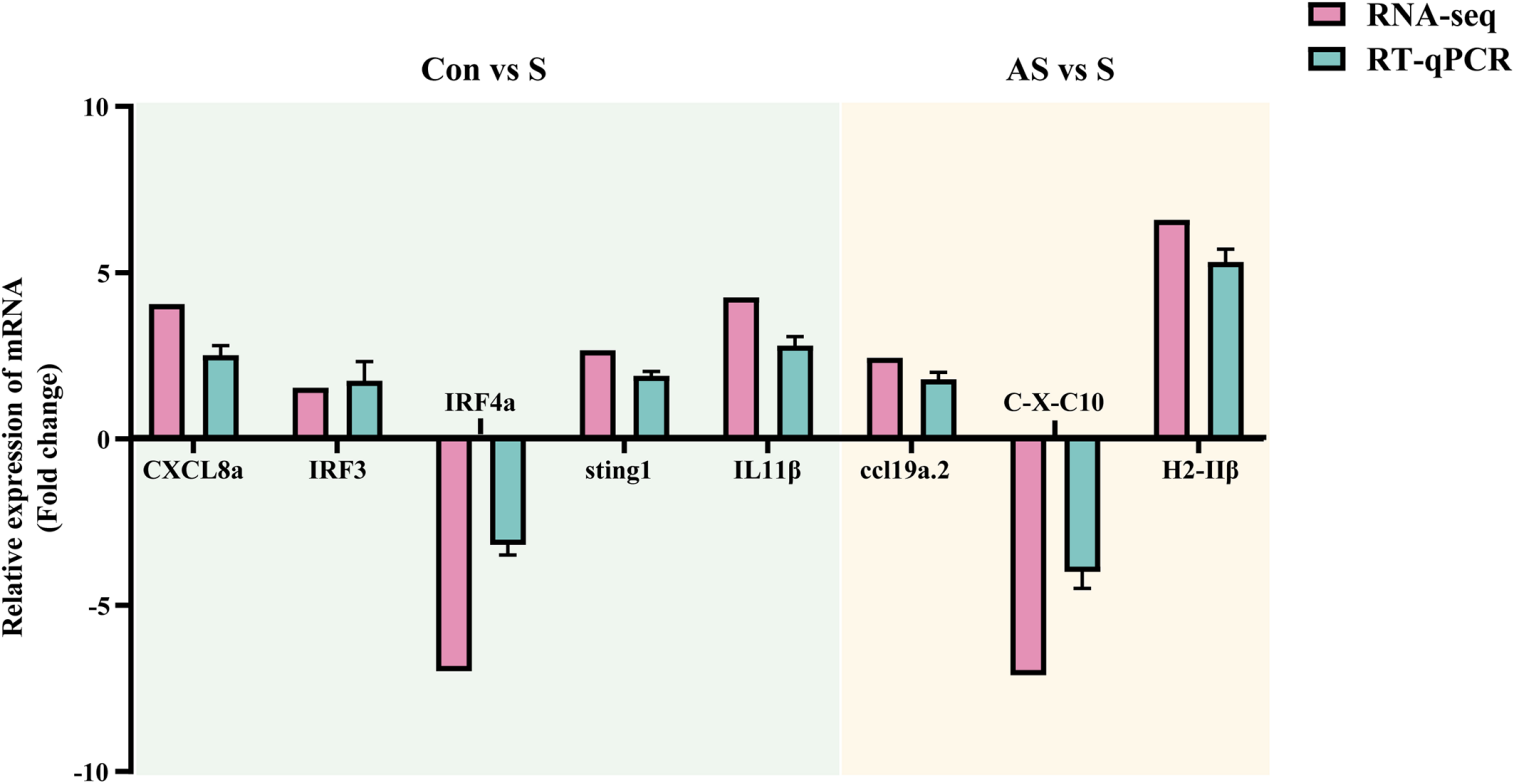
Validation of randomly selected DEGs by using RT-qPCR. The light blue area showed differentially expressed genes between control group Koi and symptomatic infected Koi. The light yellow area showed differentially expressed genes between asymptomatic infected and symptomatic infected Koi. Three biological replicates were set up for each sample.

To verify the validity and accuracy of transcriptome sequencing, significant DEGs were detected by RT-qPCR. CXCL8a, Sting, IRF3, IRF4a, IL11β C-X-C motif chemokine 10-like, H-2 class II histocompatibility antigen, E-S beta chain and ccl19a.2 were selected for RT-qPCR. CXCL8a, Sting, IRF3 and IL11β were up-regulated in the comparison groups between control and symptomatic Koi, and IRF4a was down-regulated in this comparison group. In addition, in the asymptomatic infected Koi vs symptomatic infected Koi, ccl19a.2 and H-2 class II histocompatibility antigen, E-S beta chain were up-regulated, and C-X-C motif chemokine 10-like was down-regulated. The results showed that the relative expression levels of DEGs were consistent with the RNA-seq data, demonstrating the validity of transcriptome analysis (**Figure 13**).

## 4 Discussion

The mortality of KSD ranges from 5% to 100%, which may related to the virulence of CEV. The virulence of CEV strains ranges from highly pathogenic, causing death within a few days, to weakly pathogenic, causing subclinical or persistent infections with low levels of morbidity and mortality. Moreover, the subclinical or persistent infections can re-activated as acutely infection at some conditions, such as temperature changes according to seasonal changes. In this study, CEV was detected in tissues from acutely infected and asymptomatic Koi, while was not detected in control Koi by PCR. There were significant differences in pathology and viral load between asymptomatic and acutely infected Koi. The activity of immune-related enzymes, the difference changes of mucus cells and the expression of interleukins verified the differences in immune response of Koi under different infection conditions.

CEV is a double stranded DNA virus belonging to the family Poxviridae, and phylogenomic analysis has confirmed that CEV and the salmon gill poxvirus (SGPV) were sister species, and together form the deepest branch of the Chordopoxvirinae within the family Poxviridae (5). Although poxviruses were regarded as non-latent, experimental inoculation of poxviruses in mammalian cell lines has indicated that long-term latency presumably can occur (30, 31), and the poxviruses remained latent in the transfected immune cells for periods of several months to longer than 2 years (31, 32). Recently, CEV was detected in gill tissue of Koi imported from Asia to Germany, although the Koi showed no clinical symptoms (20). This might be a stress-related reactivation of a persistent CEV infection, showing a potential dispersal route of CEV, which also reported in fowl pox infections before (33). However, the current study did not pay close attention to the difference between asymptomatic and symptomatic CEV infection. Moreover, the understanding of the molecular correlates of the severity of these infections was still unknown. This study was distinguished between asymptomatic and symptomatic Koi by clinical signs, pathology changes, nested PCR and RT-qPCR. The results showed that the presence of CEV in asymptomatic Koi, and the proportion was as high as 56.5%, but the viral load of CEV was considerably low in tissues. This was basically consistent with the detection of CEV infection in asymptomatic Koi reported by Adamek (20, 34). Enzyme activity, activity of immune-related substances and expression of immune-related gene were compared at different stages during CEV infection. There were significant differences between acutely infected and asymptomatic Koi.

As a multifunctional organ, gills not only participate in the respiration of fish, but also play a significant role in metabolism, ammonia nitrogen and osmotic pressure balance (27). In addition, as one of the organs in direct contact with the external environment, gills were also the first line of defense against pathogens and play an important role in immunity (22). The main pathological changes during CEV infection were limited to the gills. The Koi infected with CEV exhibited floating heads, which is thought to be possibly due to hypoxia caused by damage to gill tissue. However, study had reported that the hemoglobin levels is rising during CEV infection (23), and fish have evolved this mechanism to cope with hypoxia (35). Severe impairment of osmoregulation was considered to be the potential cause of death of infected CyHV-3 (36). Since the Koi infected with CEV causes severe gill injury, whether severe impairment of osmoregulation was also considered to be the potential cause of death of infected CEV. The mortality rate of common carp of CEV infection can be decreased using a long-term curative 0.5% salt water bath (37), and the osmotic balance associated with hyponatremia and hyperammonemia during CEV infection have been demonstrated (24), suggesting that osmotic pressure imbalance may also be a potential cause of CEV pathogenesis (23, 24). Ammonia poisoning has a suppressive effect on innate immunity and affects the composition of immunoglobulin (38). There was also suggested that CEV could have direct immunosuppressive effect on host, but this need more investigations to be confirmed (1, 39).

The fish immune system is divided similarly to that of mammals into an innate (non-specific) and an adaptive (specific) part (40). Innate immunity is phylogenetically older than adaptive immunity and it is the first line of defence against spreading of the pathogen after its invasion into the body (40, 41). As the first line of defense against pathogen invasion, gills contain mucus cells and can secrete a large amount of mucus (28). The mucus contains a variety of active substances, such as mucopolysaccharides, glycoproteins, immunoglobulin and various enzymes, which play an important role in immune response (42). In this study, it was found that mucus cells migrated outward from the base of the branchial lamella during CEV infection. This migration was probably caused by the proliferation of the base cells of the branchial lamella, because the branchial lamella seriously proliferated during CEV infection. In addition, the number of mucus cells increased, especially when the pathological injury was more severe. This suggests that mucosal immunity plays an important role in the resistance to CEV invasion. Malondialdehyde (MDA) is a product of lipid peroxidation caused by free radicals in the body; it can polymerize with protein molecules in cells and impair control cell function by altering protein structure. MDA activity was elevated during CEV infection, and the difference was significant in Koi with clinical symptoms, suggesting that CEV infection can cause oxidative damage. The antioxidant enzymes CAT and GSH were elevated during CEV infection, glutathione peroxidase (GSH-Px) and catalase (CAT) can decompose toxic hydrogen peroxide (H_2_O_2_) into H_2_O and O_2_, which play an important role in enhancing the defense ability of phagocytes and the immune function of the body (43). AKP and ACP increased in the process of CEV infection, and the difference was significant in Koi with clinical symptoms. ACP and AKP are important phosphatases in the body, playing an important role in metabolism and immunity (44). Lysozyme can enhance the immune function of the body and play an important role in fighting against the invasion of various pathogens (45). Although lysozyme showed an increasing trend, there was no statistically significant difference. Higher expression of immune genes during the CEV infection may have inhibited viral replication and mount an antigenic adaptive response. Studies have shown that genes related to immune response were highly expressed after CEV infection, and these genes were basically involved in innate immune response (25). The results indicated that TNF-α and IL-10 were up-regulated in CEV infected koi in our study, which consistent with the previous report (25). In addition, immune-related enzyme activity also increased after CEV infection, such as lactic acid and alkaline phosphatase (23). This was consistent with our study, indicating that the enhanced innate immune response in the process of CEV infection can prevent the further spread of CEV in the body.

Besides innate immunity, fish immune system also has adaptive immunity, and immunoglobulin plays an important role as the main part of adaptive immunity (46). Unlike innate immunity, both immunoglobulin IgM and IgT were significantly decreased after CEV infection, and the down-regulation of immune-related genes IgM, CD4+ and up-regulation of IL10 further explained the inhibitory effect on adaptive immunity. In 2021, a paper reported that immune-related genes CD4, TCRα2 and IgM were significantly down-regulated in gill tissue after CEV infection, which may lead to immunosuppressive effects in the body (24). Transcriptome analysis showed that innate immunity was activated and acquired immunity was suppressed in early Vibrio alginolyticus infection of Larimichthys crocea. Innate immunity played an important role in resistance to early Vibrio alginolyticus infection of larimichthys crocea (47). Based on the above studies and analyses, adaptive immunosuppression may be induced during CEV infection, which may contribute to the immune escape of CEV and causing more serious damage to the body.

Transcriptome sequencing can effectively reflect the differential expression of different genes and play an important role in the study of pathogenic mechanism. Transcriptome analysis revealed the immune mechanism of CyHV-3 resistant carp strains, which laid a foundation for the screening of CyHV-3 resistant carp strains (48). Transcriptome analysis of the gill of Atlantic salmon (Salmo salar L.) affected by amoeba gill disease (AGD) reveals the role of tumor suppressor gene p53 in the pathogenesis of AGD, providing a good scientific basis for further prevention and treatment of AGD (49). Our studies have shown that CEV infection causes severe damage to gill tissue, in addition to dramatic changes in gill immune-related function, transcriptome analysis was performed on gill tissues of control, asymptomatic and clinically symptomatic Koi, and a large number of differentially expressed genes were annotated. GO analysis showed that in the comparison group of asymptomatic Koi vs symptomatic Koi, in biological function, the GO term enrichment of immune response and immune system response was the most significant and the number of differentially expressed genes was the largest, this suggests that immune function plays an important role in the progression from asymptomatic to symptomatic during CEV infection. In addition, the comparison group of control Koi vs symptomatic Koi showed a lot of differentially expressed immune-related genes. There was a total of 19 co-expressed genes between asymptomatic Koi vs symptomatic Koi and control Koi vs symptomatic Koi, including 6 up-regulated genes and 13 down-regulated genes. Up-regulated genes include cxcl8a, ccl19a.1, sting1 and tumor necrosis factor-like, and down-regulated genes include chemokines, such as C-C motif chemokine 8-like, C-C motif chemokine 20-like, C-C motif chemokine 25-like. CXCL8, also called interleukin-8, was a typical CXC chemokine that plays a key role in promoting inflammation (50). STING1 (also known as STING or TMEM173) was found to play a fundamental role in the production of type I interferons (IFNs) and pro-inflammatory cytokines in response to DNA derived from invading microbial pathogens or damaged hosts by activating multiple transcription factors (51). In mammals, the main target cells of CC subfamily chemokines are monocytes, macrophages and lymphocytes, but the specific chemokines of CC subfamily in fishes have not been determined (52). A total of nine genes were differentially expressed only in AS vs S group, including three up-regulated genes and six down-regulated genes. These differentially expressed genes were important in the process of CEV infection, especially the up-regulated genes H-2 class II histocompatibility antigen, E-S beta chain and ccl19a.2, those genes may the marker gene that makes Koi develop from asymptomatic to clinically symptomatic. KEGG enrichment analysis showed that many immune-related pathways were involved in the regulation of these genes, and NOD-like receptor signaling pathway, Cell adhesion molecules (CAMs), C-type lectin receptor signaling pathway, Toll-like receptor signaling pathway is the main enrichment pathway. NOD-like receptors, Toll-like receptors (TLRs) and C-Lectins are principal receptors of pattern-recognition receptors (PRRs), and the aim was to recognize pathogen-associated molecular patterns (PAMPs) and damage-associated molecular patterns (DAMPs) resulting in mobilization of downstream signaling responses leading to inflammation, microbial destruction and eventually activation of adaptive immune responses, playing an important role in resisting the invasion of pathogens (53, 54). These pathways may play an important role in immune response and show significant different in the course of CEV infection. How they regulate the changes in immune response, especially from asymptomatic to symptomatic infection, will be an interesting topic and worthy for further study.

## Data availability statement

The datasets presented in this study can be found in online repositories. The names of the repository/repositories and accession number(s) can be found below: PRJNA925573 (SRA).

## Ethics statement

The animal study was reviewed and approved by Animal Care and Use Committee of Sichuan Agricultural University.

## Author contributions

PO, YR, YZ, QL, DC and LY participated in material preparation and experimental investigation; QL, YG, HG, JF and HD performed research and analyzed results; YR and XH analyzed RNA-Seq data; WL prepared the experiment materials; ZC and GS developed methodology and discussed results; YR and LY designed research, wrote the manuscript and supervised the study. All authors have read and agreed to the published version of the manuscript.
